# Applications of Catechins in the Treatment of Bacterial Infections

**DOI:** 10.3390/pathogens10050546

**Published:** 2021-05-01

**Authors:** Meishan Wu, Angela C. Brown

**Affiliations:** Department of Chemical and Biomolecular Engineering, Lehigh University, Bethlehem, PA 18015, USA; mew319@lehigh.edu

**Keywords:** catechins, antibacterial, anti-virulence, antibiotic resistance, toxin

## Abstract

Tea is the second most commonly consumed beverage worldwide. Along with its aromatic and delicate flavors that make it an enjoyable beverage, studies report numerous health advantages in tea consumption, including applications in antimicrobial therapy. The antimicrobial properties of tea are related to catechin and its derivatives, which are natural flavonoids that are abundant in tea. Increasing evidence from in vitro studies demonstrated antimicrobial effects of catechins on both gram-positive and gram-negative bacteria, and proposed direct and indirect therapeutic mechanisms. Additionally, catechins were reported to be effective anti-virulence agents. Furthermore, a number of studies presented evidence that catechins display synergistic effects with certain antibiotics, thus potentiating the activity of antibiotics in resistant bacteria. Despite their numerous beneficial properties, catechins face many challenges in their development as therapeutic agents, including poor absorption, low bioavailability, and rapid degradation. The introduction of nanobiotechnology provides target-based and stable delivery, which enhances catechin bioavailability and optimizes drug efficacy. As further research continues to focus on overcoming the unresolved challenges, catechins are likely to see additional promising applications in our continual fight against bacterial infections.

## 1. Introduction

Antibiotic-resistant bacterial infections are one of the most significant global threats to public health. In a 2019 report released by the United States Centers for Disease Control and Prevention, at least 2.8 million people per year acquire an antibiotic-resistant infection, resulting in more than 36,000 deaths in the United States [1]. New antibiotics and alternative therapeutic strategies to treat the increasing numbers of these infections are urgently needed. 

**Figure 1:**
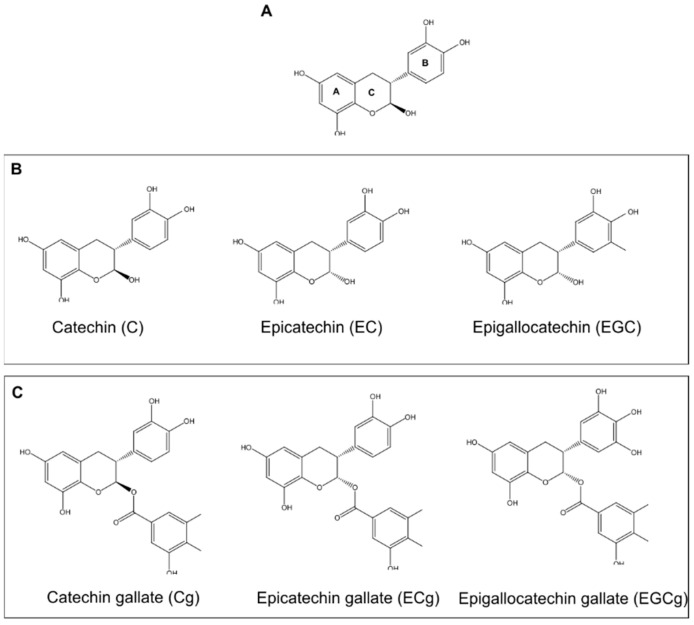
Catechin Structures. (**A**) General Catechin Structure. The structure of catechin consists of two benzene rings (A and B) connected by a dihydropyran heterocyclic ring (C), containing a hydroxyl on carbon 3. (**B**) Structures of commonly used nongalloylated catechins. (**C**) Structures of commonly used galloylated catechins.

One natural compound that has emerged as a potent substitute in treating antibiotic-resistant strains of bacteria is catechin [2]. Catechin is a natural tea polyphenol (TPP) abundant in green tea, accounting for 30–42% of the solid dry weight of brewed tea. Catechins are comprised of two benzene rings (A- and B-rings), linked by a dihydropyran heterocycle (C-ring), which contains a hydroxyl moiety on carbon 3 [3,4]. The catechins are often classified into nongalloylated and galloylated groups, based on the absence or presence of a gallate moiety (Figure 1) [3,4]. 

Catechins are demonstrated to possess a number of promising bactericidal effects on both gram-positive and gram-negative bacteria, including multidrug-resistant strains. Additionally, these molecules are shown to inhibit virulence factor activity, particularly toxins, thus reducing the pathogenicity of certain bacteria. Importantly, certain catechins were observed to synergize the activity of traditional antibiotics. Unfortunately, the therapeutic use of catechins has been limited by several issues, including a short shelf-life, limited bioavailability, and low stability. However, the use of novel nanocarriers has helped to overcome these limitations, providing renewed promise of the therapeutic potential of catechins in the fight against antibiotic-resistant bacterial infections. In this review, we highlight the many reported uses of catechins to treat bacterial infections and describe biotechnological approaches to overcome the limitations of the molecules. We highlighted those papers most relevant to the topic to provide an overview of the therapeutic possibilities of these molecules.

## 2. Antibacterial Properties of Catechins

The antimicrobial potential of tea is a point of interest for repurposing natural compounds in biochemistry. Experiments conducted to investigate the antibacterial property of tea go back more than one hundred years, when J. G. McNaught observed that *Bacillus typhosus* diminished greatly in number when immersed in cold tea [5]. Since then, the antibacterial activity of catechins was observed in many different species, both gram-negative and gram-positive (Table 1). In recent years, interest in these antibacterial properties has increased as resistance to traditional antibiotics continues to expand. 

Jeon et al. used epigallocatechin gallate (EGCg) and green tea extracts (GTE) to inhibit the growth of two gram-negative species, *Pseudomonas aeruginosa* and *Escherichia coli*. In this study, a total of 22 strains were isolated from skin wounds, and two reference strains were used as controls. Five of the collected *P. aeruginosa* strains showed multidrug resistance, and nine *E. coli* strains showed resistance to at least one antibiotic, demonstrating the urgency for alternative bactericidal strategies to overcome resistance to current antibiotics. In this set of experiments, EGCg, at concentrations greater than 0.4 mg/mL, was found to inhibit the growth of all strains of *E. coli,* and an even lower concentration range (0.2–0.4 mg/mL) was found to inhibit the growth of all strains of *P. aeruginosa*. The level of EGCg effectiveness in these strains differed from the pattern of antibiotic resistance, suggesting that EGCg could be used as an alternative strategy to kill bacteria that are resistant to one or more antibiotics [6]. 

The effects of catechins including EGCg on gram-positive bacteria were reported to be even stronger. Bai et al. explored the inhibitory effects of several catechins on *Streptococcus mutans*, a biofilm-forming, gram-positive bacterium commonly found in oral cavities. Each of the tested molecules, with the exception of catechin (C), had some inhibitory activity against the bacteria. EGCg demonstrated the strongest activity, with a minimum inhibitory concentration (MIC) of 0.125 mg/mL. At a slightly higher concentration (0.2 mg/mL), EGCg was able to prevent biofilm formation efficiently. Field emission-scanning electron microscopy (FE-SEM) was used to study the morphology of *S. mutans* cells in both planktonic and biofilm forms, and images revealed that EGCg treatment caused irreversible damage to the cytoplasmic membrane of the *S. mutans* cells [7].

Such evidence of membrane damage led to one of two leading hypotheses to describe the antibacterial mechanism of catechins. In the membrane-disruption hypothesis, catechins intercalate into the lipid bilayer, leading to lateral expansion and membrane disruption. An alternative hypothesis posits that catechins oxidize in the cell culture medium, generating hydrogen peroxide, which leads to damage in DNA and protein oxidation. Evidence supporting each of these hypotheses is described below. 

### 2.1. Lateral Expansion/Membrane Disruption

**Figure 2:**
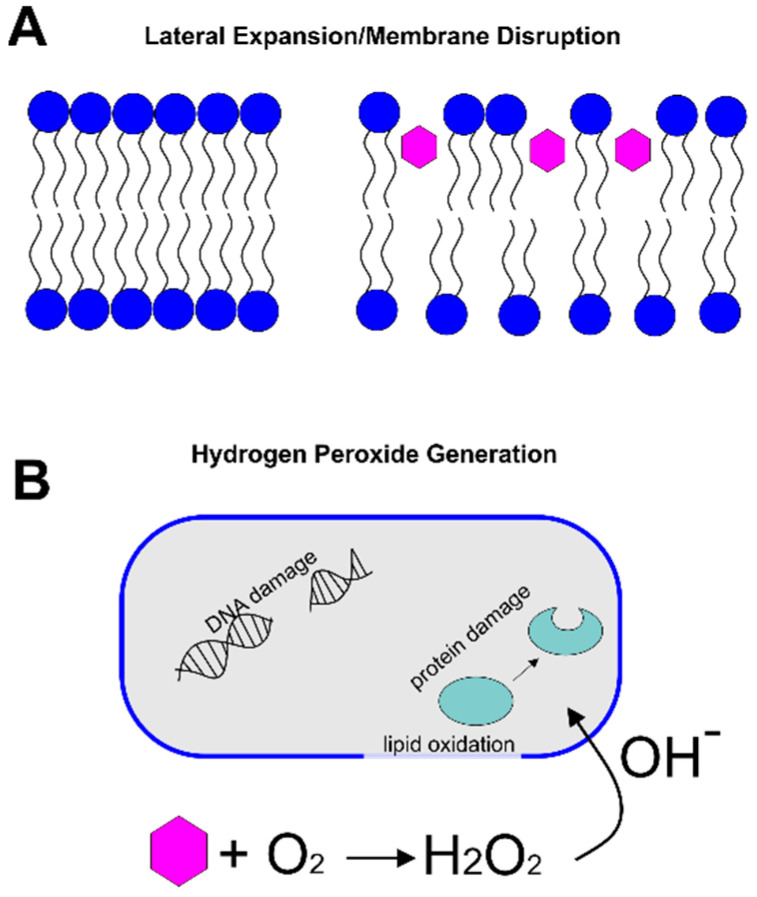
Antibacterial Mechanisms of Catechins. (**A**) In the lateral expansion/membrane disruption hypothesis, catechins (pink hexagons) intercalate into the lipid bilayer, increasing the spacing between lipids and the resulting membrane permeability. (**B**) In the hydrogen peroxide generation mechanism, catechins (pink hexagon) react with dissolved oxygen to produce hydrogen peroxide and hydroxyl radicals. These radicals cause lipid oxidation and DNA/protein damage inside the cell.

Catechins have long been known to have a strong ability to partition into lipid bilayers of various compositions [8,9,10]. Because of this behavior, it has been hypothesized that the partitioning of catechins within the membrane of bacterial cells decreases lipid packing and increases lateral expansion of the membrane, resulting in a decreased barrier activity of the membrane (Figure 2A). To explore the bactericidal mechanism of catechins and their impacts on the bacterial cell membrane, several computational and experimental methods were developed to study the specific interactions between catechins and the lipid bilayer. 

Sirk et al. used molecular dynamics simulations to study the interactions between several catechins and a mixed bilayer composed of 1-palmitoyl-2-oleoyl-glycero-3-phosphocholine (POPC) and palmitoyl-2-oleoyl-glycero-3-phosphatidylethanolamine (POPE). The simulations showed that binding to the lipid headgroups near the bilayer surface (adsorption) was the initiating step, and some of the molecules, including C, epicatechin (EC), and EGCg, penetrated deeper into the bilayer interface (absorption) [11]. In general, molecules without the gallate group (C and EC) were able to penetrate better into the lipid bilayer, which suggests that the catechin chemistry regulates their membrane partitioning. The authors then looked at hydrogen bonding between the catechins and membrane lipids, specifically between the hydroxyl groups of the catechin and the oxygen atom of the lipids, or between the oxygen atom of the catechins and the ethanolamine groups of POPE. The simulation demonstrated that the surface binding of the catechins was determined by the ability of each molecule to form hydrogen bonds with the lipids [11]. EGCg can form five hydrogen bonds, more than the other catechins [11], providing a possible explanation for previous observations that EGCg is the most biologically active catechin [12]. The incorporation of the catechins led to an expansion and increase in the lateral area of the membrane. Each EGCg molecule occupied an average area of 1.80 nm^2^, approximately three times the area occupied by a lipid in the absence of EGCg [13].

Experimental studies into the mechanisms of EGCg-mediated membrane disruption used model membrane systems to probe these interactions more closely. Using giant unilamellar vesicles (GUVs), Tamba et al. employed phase-contrast fluorescence microscopy to study how EGCg influences membrane integrity. Calcein-encapsulated GUVs were prepared using egg phosphatidylcholine (PC). At a low concentration of EGCg (≥30 μM), calcein leakage was observed, with all of the intraliposomal calcein being released within 6 s; complete, irreversible disruption of the GUVs was observed within 5 min. At lower concentrations of EGCg, the authors observed reversible shape changes, indicating an increase in the lateral area of the membrane. They hypothesized that with increasing concentrations, EGCg partitioning into the membrane continues to expand the lateral area, thus continually decreasing the activation energy for the irreversible membrane disruption process [9]. 

To investigate the disparity between the effectiveness of catechins in treating gram-positive and gram-negative bacteria, Kajiya et al. manipulated the charge of the model membrane by incorporating 10% phosphatidylserine (PS, negatively charged), dicetyl phosphate (DCP, negatively charged), or stearylamine (SA, positively charged) to egg PC liposomes. The authors found that the amount of epicatechin gallate (ECg) and EGCg incorporated into the negatively charged liposomes (containing either PS or DCP) was significantly reduced relative to that incorporated into neutrally charged (PC) or positively charged (containing SA) membranes [14]. This finding is consistent with another report that negatively charged lipids, including PS and DCP, inhibited EGCg-mediated membrane disruption [15]. The authors suggested that the low permeability of catechins in negatively charged membranes might explain the relative resistance of gram-negative bacteria to the effects of catechins [14,15]. 

Together, these structure-function studies demonstrated that the affinity and partitioning behaviors of catechins depend on (1) the structural and stereochemical attributes of the catechins and (2) the physical properties of the membrane, particularly the charge. While some trends were observed, several exceptions exist, suggesting that these properties may not fully regulate the antibacterial activity of the catechins. Importantly, in all of the experiments and simulations conducted to date, non-bacterial lipids, including PC and PS, were used, making it difficult to translate findings to living bacterial cells, which are composed primarily of other lipids, such as phosphatidylethanolamine (PE), phosphatidylglycerol (PG), and lipopolysaccharide (LPS). In addition, the effects on catechin-mediated membrane disruption of the physical state of the studied membranes and the environmental conditions are not well studied. Future work to focus on the specific interactions between catechins and more relevant bacterial membranes could alleviate these problems. 

### 2.2. Hydrogen Peroxide Generation

An alternative explanation, related to the oxidation of catechins, was proposed to explain the observed antibacterial effects (Figure 2B). Arakawa et al. first demonstrated that, by using peroxalate chemiluminescence and electron spin resonance, the reaction between catechins and dissolved oxygen in the culture led to the generation of reactive oxygen in the form of hydrogen peroxide [16]. They then showed the relationship between the generated hydrogen peroxide concentration and bacterial viability by adding catalase to the culture to facilitate the decomposition of hydrogen peroxide. The authors observed that as the concentration of catalase introduced into the culture medium was increased, the bactericidal action of the catechins decreased, thus supporting the hypothesis that hydrogen peroxide contributes to the antibacterial mechanism of catechins [16]. 

The corrosive feature of hydrogen peroxide is the result of the Haber-Weiss reaction, in which superoxide radicals lead to the production of short-lived, yet highly reactive, free hydroxyl radicals [17]. The resulting oxidation of amino acid residues can modify protein backbones and alter the side-chain structure, causing DNA damage and oxidation of essential cellular components such as the cell membrane and organelles [17]. Imlay and Linn discussed two modes by which hydrogen peroxide may kill *E. coli* K12 cells. At concentrations below 3 mM, DNA damage occurs in cells with active metabolism. At higher concentrations, a non-specific, dose- and time-dependent effect is observed, which does not require that cells be metabolically active [18]. 

This pro-oxidative activity of catechins was utilized by Shishido et al. to develop a photoirradiative system to kill bacterial cells. The authors hypothesized that photoirradiating (+)-catechin, which has minimal bactericidal activity alone, with blue light (400 nm) would result in the formation of hydrogen peroxide and the generation of hydroxyl radicals that lead to bacterial cell death. The authors observed that photoirradiation of the catechin resulted in a reduction of *Staphylococcus aureus* viability in a time-dependent manner. Within 20 min, a more than five-log reduction in viability was observed [19]. 

## 3. Anti-Virulence Properties of Catechins

**Figure 3:**
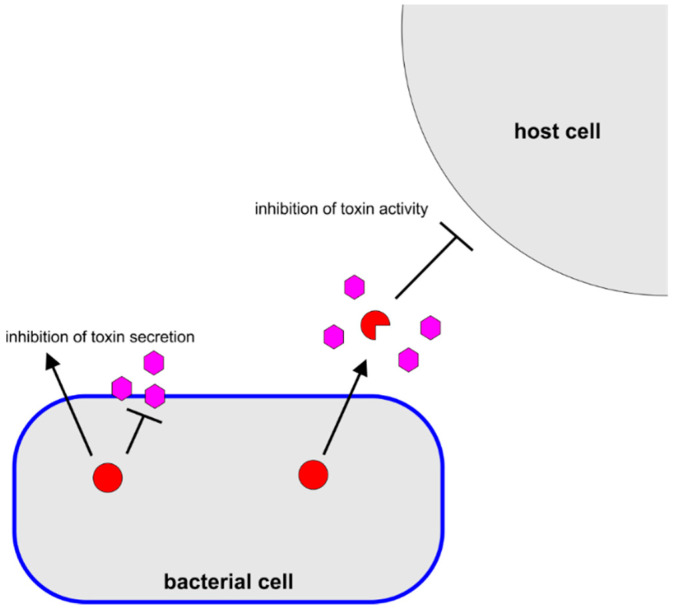
Antivirulence Mechanisms of Catechins. Catechins (pink hexagons) were observed to inhibit bacterial toxin (red circle) secretion or alter toxin conformation, thereby inhibiting activity.

Pathogenic bacteria often express a number of so-called virulence factors, which somehow damage or otherwise affect the host to enhance the virulence of the organism [20,21]. Inhibition of the activity of these virulence factors, an “anti-virulence approach” has gained interest in recent years as an alternative to traditional antibiotics [22,23,24]. In addition to their antibacterial properties, catechins have been reported to exhibit a number of anti-virulence activities, particularly inhibition of toxin activity (Figure 3). Often, these anti-virulence activities are observed at lower concentrations than their antibacterial activities (Table 2). It was proposed that because anti-virulence approaches do not exhibit the same selective pressure as antibacterial approaches, resistance to the drug may develop more slowly [25,26]. Therefore, the identification of these anti-virulence properties of catechins has received much attention. 

In one of the first reports of the anti-toxin activity of tea, Okubo et al. observed that tea inhibited the hemolytic activity of the *S. aureus* α-toxin and the *Vibrio parahaemolyticus* thermostable direct hemolysin; coffee had very little inhibitory effect [27]. Subsequent work by this group demonstrated that it was the presence of specific catechins, in particular ECg and EGCg, that endowed this anti-toxin activity to the tea [36,37]. 

Cherubin et al. evaluated the effectiveness of polyphenolic compounds from grape extract, including catechins, as an inhibitor for cholera toxin (CT). CT is an AB5 toxin, where the A subunit (CTA) is the active, catalytic component, and the B subunit (CTB) regulates binding to the host cell. Of the 20 isolated polyphenolic compounds, EGCg was determined to be the most effective compound in inhibiting CT activity and was selected for further analysis. EGCg reduced the binding of CTB to host cells by approximately 60%. In combination with procyanidin B2 (PB2), binding of CTB to host cells was reduced by 70% [28]. The authors thus concluded that these two compounds were likely responsible for the grape extract-mediated inhibition of CT binding [38]. The authors demonstrated that EGCg interacts directly with CTB, rather than with the cell surface, changing the conformation of the subunit and thus inhibiting cell binding. The authors also found that EGCg inhibits the activity of the CTA subunit, reducing the cyclic adenosine monophosphate (cAMP) response of the host cells to less than 30% of the control response. EGCg was further found to inhibit the activity of several other AB5 toxins, including ricin, *P. aeruginosa* exotoxin A (ETA), and diphtheria toxin (DT). 

Sugita-Konishi et al. explored the ability of six different catechins to inhibit the production and extracellular release of the AB5 toxins, Stx1 and Stx2 (also called Vero toxin 1 and 2 (VT1, VT2)), by the enterohemorrhagic *E. coli* (EHEC) O157:H7 strain. The authors observed that EGCg and gallocatechin gallate (GCg), but none of the other tested catechins, decreased the amount of Stx1 and 2 released into the supernatant. Additionally, both EGCg and GCg increased the amount of Stx1 located on the surface of the EHEC cells but had no effect on the amount of cell-surface associated Stx2. The release of Stx1 and 2 into the culture supernatant was completely inhibited by EGCg at concentrations of 250 μg/mL and 500 μg/mL, respectively [29]. As AB5 proteins, the Stx toxins are synthesized in the cytoplasm and assembled in the periplasm before release [39]. The authors observed that EGCg and GCg also inhibit the release of another periplasmic protein, maltose binding protein (MBP), and thus hypothesized that the catechins act on the secretion machinery rather than on the toxins themselves [29]. A subsequent study demonstrated that GCg and EGCg have additional inhibitory effects on Stx1 after release, preventing its cytotoxicity against Vero cells [30].

*Bacillus anthracis* secretes a toxin, anthrax toxin (AT), which consists of a lethal factor (LF), a protective antigen (PA), and an edema factor (EF) [40]. After PA binding to the host cell surface, it becomes capable of binding either LF or EF. The LF-PA complex is endocytosed, and in the late endosomal compartment, LF displays proteolytic activity against the mitogen-activated protein kinase kinase (MAPKK) family of proteins [40]. Dell’Aica et al. found that EGCg inhibits this proteolytic activity of LF and, as a result, protects macrophages from AT-mediated cell death. Pretreatment with EGCg similarly protected Fischer 344 rats from AT-mediated death [31]. 

As part of its virulence arsenal, *Streptococcus pneumoniae* produces pneumolysin (PLY), a thiol-activated, pore-forming cholesterol-dependent cytolysin (CDC) that disrupts host cell function [41]. Song et al. applied in vitro and in vivo experiments along with computational modeling to determine that EGCg inhibited both the hemolytic and cytolytic effects of PLY in a concentration-dependent manner by preventing oligomerization of PLY monomers to form a pre-pore complex. The authors also demonstrated that EGCg inhibits the activity of sortase A (SrtA), a transpeptidase that mediates the covalent attachment of specific proteins to the bacterial cell wall [42]. Finally, the authors demonstrated that with this dual mode of action, EGCg was able to prevent *S. pneumoniae* colonization in a mouse model [2]. 

EGCg was also reported to inhibit the activity of another CDC, listeriolysin O (LLO), produced by the gram-positive, intracellular bacterium, *Listeria monocytogenes*. After the bacterium is phagocytosed, LLO disrupts the phagosomal membrane, enabling bacterial escape to the cytosol [43]. Kohda et al. demonstrated that EGCg bound to the cholesterol-binding site of LLO, thus inhibiting the ability of LLO to disrupt the phagosomal membrane. As a result, the bacteria were unable to escape the phagosome, and intracellular growth was inhibited [32]. 

We and others observed strong anti-toxin activity by the galloylated catechins against the leukotoxin (LtxA) produced by *Aggregatibacter actinomycetemcomitans*. LtxA is a member of the repeats-in-toxin (RTX) family of toxins, and specifically kills human immune cells, thus limiting the host immune response to infection. Kawashima observed that galloylated catechins (catechin gallate (Cg), GCg, ECg, and EGCg) inhibited LtxA toxicity against HL60 cells, a myeloblast cell line [34]. We studied the mechanism of this activity and found that galloylated catechins significantly change the toxin’s secondary structure. With this altered structure, LtxA was unable to recognize and bind cholesterol on the host cell membrane, an essential component of the toxin’s mechanism [33]. We also observed that EGCg has large effects on LtxA release. When *A. actinomycetemcomitans* was treated with EGCg at a sub-inhibitory concentration (5 μg/mL), more LtxA was produced; however, most of the toxin remained in association with the bacterial cell surface rather than being secreted into the supernatant [44,45]. 

Like many bacterial toxins, LtxA is released in both a “free” form (as a single protein, released into the extracellular environment) [46], as well as in association with outer membrane vesicles (OMVs) [47,48]. OMVs are spherical lipid entities, commonly released by gram-negative bacteria derived from the bacterial outer membrane consisting of an outer layer of LPS [49]. OMVs were recognized to play a crucial role in mediating pathogen-host interactions, aiding bacteria in adapting to hostile environments, and modulating the host cell immune response, among other functions [49,50,51]. We and others demonstrated that secreted LtxA resides on the surface of *A. actinomycetemcomitans* OMVs and is delivered to host cells in an active form [47,48,52]. Saito et al. demonstrated that catechins inhibit the cytotoxicity of *A. actinomycetemcomitans* OMV-associated LtxA [35]. However, we recently discovered that EGCg-treated *A. actinomycetemcomitans* produces OMVs containing approximately six-fold more LtxA than does untreated *A. actinomycetemcomitans*, a finding that may be due the increased association of LtxA with the bacterial cell surface in the presence of EGCg [45]. 

## 4. Potentiation of Antibiotics 

In addition to their promising use as antibiotics or anti-virulence molecules, catechins were proposed as potentiating factors to improve antibiotic effectiveness. Catechins, as a single compound or cocktail, were reported to increase bacterial susceptibility to β-lactam and other broad-spectrum antibiotics by a number of mechanisms, including altering cell membrane permeability to increase the uptake of antibiotics and/or by inhibiting the function of multidrug efflux pumps to limit export of the drugs.

The ability of catechins to exert synergy with penicillins in methicillin-resistant *Staphylococcus aureus* (MRSA) was demonstrated by several groups. The β-lactam antibiotics act by binding to a family of proteins called penicillin binding proteins (PBPs), which are responsible for polymerization and cross-linking of peptidoglycan [53]. When the penicillin is bound to the PBP, the ability of the enzyme to catalyze these reactions is inhibited, resulting in peptidoglycan defects [53]. MRSA encodes an alternate PBP (PBP2’), for which penicillins have a reduced affinity; as a result, MRSA is able to continue to synthesize peptidoglycan [54]. 

In one of the first reports on the beneficial properties of catechins against MRSA, Yam et al. demonstrated that a crude tea extract displayed synergy with methicillin against MRSA. The authors attributed this effect to the extract-mediated inhibition of PBP2’ production by the cells [55]. Subsequent work identified ECg as the compound responsible for this activity [56]. 

An alternative mechanism to explain this synergistic behavior was proposed by Zhao et al., in which EGCg promotes the activity of the penicillins by acting either directly or indirectly on the peptidoglycan of the cell wall. The authors found that EGCg enhanced the effects of all tested penicillins against MRSA but not against *E. coli* or any other gram-negative bacteria. In addition, they observed only additive effects between EGCg and non-penicillin antibiotics. Peptidoglycan blocked the synergistic and antibacterial effects of EGCg. As a result, the authors concluded that EGCg targets the cell wall, increasing the effectiveness of cell-wall targeting antibiotics [57]. 

**Figure 4:**
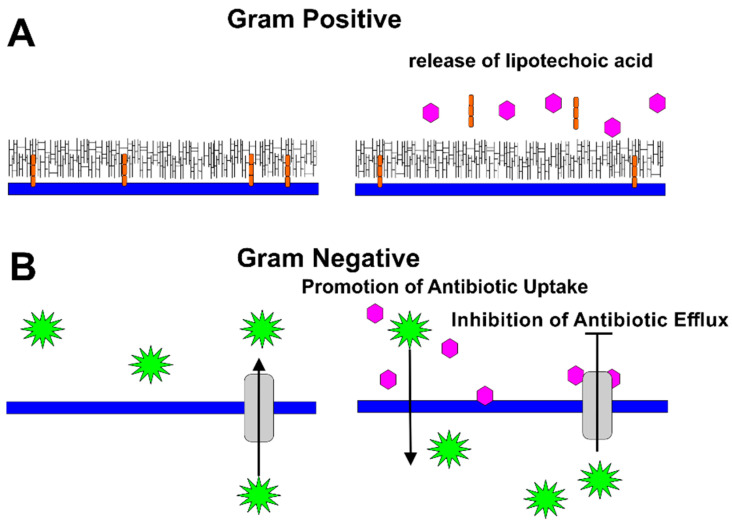
Mechanisms by Which Catechins Potentiate Antibiotics. (**A**) In gram-positive bacteria, catechins (pink) mediate the release of lipoteichoic acid (orange), weakening the cell wall of the bacteria. (**B**) In gram-negative bacteria, catechins (pink) disrupt the packing of the outer membrane, increasing permeability of the antibiotics (green). In addition, catechins were observed to inhibit the activity of bacterial efflux pumps, thus limiting antibiotic export by the bacteria.

In subsequent work, Stapleton et al. demonstrated that the gallate moiety is essential for the synergistic activity of catechins and penicillins, including flucloxacillin, imipenem, and meropenem against MRSA [58]. This group later observed that catechins with a gallate moiety (ECg and EGCg) increased the sensitivity of *S. aureus* to oxacillin, as well. Nongalloylated catechins did not have a similar effect; however, the nongalloylated catechins (EC and EGC) increased the ability of ECg and EGCg to decrease the MIC of oxacillin against MRSA [59]. The authors later discovered that ECg reduces binding of penicillin to PBPs and the resulting degree of peptidoglycan cross-linking; they also observed the release of lipoteichoic acid from the cell membrane [60]. Thus, the authors proposed that ECg reduced β-lactam resistance in MRSA, either by binding to PBPs in a noncompetitive mechanism, or by intercalating into the cytoplasmic membrane and promoting the displacement of lipoteichoic acid from the membrane [60] (Figure 4A).

Synergistic effects of catechins with certain antibiotics were also reported in several gram-negative bacteria (Figure 4B). 

*Porphyromonas gingivalis* is a gram-negative bacterium recognized as a “keystone pathogen” in chronic periodontitis [61]. Fournier-Larente et al. examined the ability of green tea extract and EGCg to potentiate the antimicrobial effect of metronidazole and tetracycline, the two antibiotics most commonly used for periodontal therapy. Both GTE and EGCg exerted synergistic effects on metronidazole for the treatment of *P. gingivalis* but exhibited only additive effects on tetracycline [62]. Although the authors did not specifically investigate the mechanism of potentiation, they hypothesized that direct effects of EGCg on the bacterial cell wall might be responsible for the observed behavior [62]. 

*P. aeruginosa* is an intrinsically multidrug resistant (MDR) organism responsible for many types of nosocomial infections [63]. EGCg was observed to display synergy with the monobactam, aztreonam, in clinical MDR strains of *P. aeruginosa*. Accumulation assays were used to show that EGCg-treated cells took up more ethidium bromide than non-treated cells due to increased permeability, decreased efflux, or both. The authors therefore hypothesized that the mechanism of the synergistic effect was an EGCg-mediated increase in the uptake and/or decrease in the efflux of aztreonam by *P. aeruginosa* [64].

Additional evidence was uncovered to indicate that EGCg inhibits bacterial efflux of antibiotics in both gram-positive and gram-negative bacteria. Roccaro et al. reported that EGCg could enhance the activity of tetracycline in resistant *Staphylococcus epidermidis* and *S. aureus* isolates expressing the tetracycline efflux pump, Tet(K). Several studies reported that efflux pumps are one of the key mechanisms responsible for antibiotic resistance [65]. Treatment with EGCg reduced the rate of tetracycline efflux by both isolates. Similar results were obtained using protoplasts, in which the cell wall had been removed, demonstrating that interactions of EGCg with the cell wall were not responsible for the synergistic effects. The authors thus concluded that synergistic effects of EGCg arise from its inhibition of Tet(K) [65]. 

The association of EGCg with bacterial efflux pumps was also demonstrated in an analysis of EGCg synergism with antibiotics against carbapenem-associated multidrug resistant *Acinetobacter baumannii*. Seventy clinical isolates of *A. baumannii* were collected, and the EGCg-treated MIC and minimum bactericidal concentration (MBC) were analyzed in the presence and absence of selected antibiotics. Results from the checkerboard assay and time-kill assay demonstrated synergism of EGCg with meropenem and carbenicillin, both of which are β-lactam antibiotics. The authors observed that EGCg had little effect on β-lactamase production; however, EGCg enhanced the effect of the efflux pump inhibitor, 1-(1-napthylmethyl) piperazine (NMP). The authors therefore concluded that EGCg might improve susceptibility to β-lactam antibiotics through the inhibition of efflux pump function [66].

While the specific mechanisms by which catechins, such as EGCg, exert synergy with antibiotics is not yet fully established, it is clear that the combination of catechins and antibiotics provides a promising approach to improve treatment of antibiotic-resistant strains of bacteria.

## 5. Strategies to Improve the Therapeutic Potential of Catechins 

### 5.1. Limitations of Catechins 

Although catechins display significant antimicrobial potential and extensive physiological benefits, as described above, some disadvantages and adverse effects have limited their therapeutic use. 

#### 5.1.1. Stability

Low stability is the primary cause for inconsistencies between laboratory experiments, whether the studies are in vitro or in vivo. Catechins undergo epimerization at various temperatures and pH conditions, resulting in the formation of polyphenolic epimers, which may have different functions than the original compound. This epimerization is accompanied by a color change from transparent to visible brown [67,68,69]. Although catechin is a known antioxidant, additional antioxidation compounds are required to maintain the transparent color of catechin solutions. The instability of catechins has placed restrictions on the bioavailability and laboratory applications of these molecules. 

TPP mixtures are more stable than pure catechin compounds due to the presence of other antioxidizing agents in the mixture. Because of its highly advertised antioxidant and antimicrobial effects, TPP is one of the most widely consumed natural compounds [70]. TPP is often provided in the form of an oral capsule, typically made with vegetable cellulose or gelatin. TPP stored in gelatin capsules has a shelf life of about two to three years at room temperature. This increased stability is due to the gelatin, which integrates with catechin to act as an effective inhibitor of epimerization or potentially to improve the therapeutic effect [71,72]. Chen et al. combined catechin and gelatin to create self-assembled nanoparticles. The authors observed that the catechins inhibited trypsin-mediated digestion of the gelatin, and the gelatin preserved the antioxidant activity of the catechins for more than three weeks of storage at room temperature [71]. Although TPP mixtures increased stability relative to pure catechins, the phenolic content of these mixtures can vary greatly, depending on the species, light exposure, growing location and altitude, season, and clonal variation [73]. 

#### 5.1.2. Specificity 

EGCg is considered one of the natural pan-assay interference compounds (PAINS), due to its catechol motif. PAINS can interfere with numerous bioassays via different mechanisms, rather than the specified target [74]. EGCg has shown promiscuous behaviors in broad bioassays, leading to extensive scientific and medical applications being granted to EGCg along with confusing and conflicting experimental results [74]. While the reactivity of EGCg may generate a protective barrier by readily attacking harmful bacteria and their virulence factors, the pro-oxidant effect of high doses can cause collapse in the mitochondrial membrane potential. Mitochondrial membrane potential is essential for energy storage during oxidative phosphorylation [75]. 

High doses of EGCg can therefore act as a mitochondrial toxin and lead to severe liver injury [76]. Popularly advertised as dietary supplement, there are increasing numbers of reported adverse events involving the intake of EGCg and concentrated GTE, which contribute to the growing concern of dietary safety and advocacy for FDA regulation. Lambert et al. reported on the dose-dependent hepatotoxic effect of EGCg in mice [77]. A high daily dose of EGCg, administered orally, resulted in cumulative liver damage [76,77]. Acute and chronic hepatitis over time can lead to fulminant liver failure, which ultimately increases mortality [78]. 

#### 5.1.3. Bioavailability 

The potential therapeutic use of catechins is also limited by the poor reported bioavailability of these molecules. In humans, the maximum catechin concentrations in plasma were measured to range from 1 to 2 μmol/L several hours after consumption; this concentration rapidly decreases to baseline levels within 24 hr [79,80,81]. This low bioavailability was proposed to arise from several factors, including low stability of the catechins within the intestines [82,83,84], limited transport of catechins across the intestine wall [85,86], and rapid metabolism and clearance [79]. 

### 5.2. Catechin Delivery Strategies

Multiple strategies, including nanotechnology, were proposed and tested to determine the most appropriate method to address these limitations and maintain optimal performance of the delivered catechins [87,88,89]. The extremely small size of nanoparticles allows them to possess unique physical properties, such as a tunable material geometry, large surface area to volume, and modifiable chemical composition [90,91]. Thus, these nanocarriers are designed precisely to deliver the encapsulated drug to targeted regions in the body [91,92]. We anticipate, with increased demonstration of the ability of these nanocarriers to overcome the known limitations of catechins, that the field will see renewed interest in the therapeutic possibilities of catechins. 

#### 5.2.1. Liposomes 

Utilizing liposomes and other lipid-based particles to encapsulate and deliver drug particles is a well-developed and widely supported method in the field of drug delivery. Liposomes are highly compatible with most therapeutic particles, possess high biocompatibility, and are readily tunable, all of which grant advantages to liposomes as a catechin delivery vehicle for the treatment of infectious disease [93,94,95]. A number of researchers demonstrated that incorporation of catechins within liposomes of various compositions increases the molecules’ stability in buffer and simulated gastric and intestinal fluids [96,97,98]. Additionally, incorporation into liposomes results in a sustained release profile [97,98], including an enhancement in concentration in the blood in a rat model [98].

Liposome-encapsulated EGCg was found to maintain its antibiotic activity against several bacterial species at a lower efficacy. This finding could be due to the slower release profile of EGCg when encapsulated within the liposomes [99]. Additionally, the surface charge of the liposomes was demonstrated to play an important role in the activity against MRSA of liposome-encapsulated EGCg [100]. The efficacy of EGCg against MRSA was found to be higher when the molecule was encapsulated in cationic liposomes than when it was delivered in a free form, or encapsulated in anionic or neutrally charged liposomes. In vitro results showed that the MICs against MRSA of EGCg as a free drug, or encapsulated in cationic, neutrally charged, and anionic liposomes, were 128, 16, 32, and 256 mg/L, respectively [100]. Subsequent in vivo tests confirmed these results, with survival rates for treatment with cationic, neutral, and anionic EGCg-loaded liposomes of 100%, 70%, and 30%, respectively [100,101]. 

#### 5.2.2. Niosomes 

Niosomes are a vesicular delivery system, composed of nonionic surfactants with molecules such as cholesterol to create non-toxic, inexpensive, stable vesicles with sustained release [87,102]. As with liposomes, the incorporation of catechins within niosomes was demonstrated to enhance the stability of the molecules [103]. In addition, this encapsulation increased catechin uptake by Caco-2 cells, a human epithelial cell line used to model intestinal uptake. The authors of this study concluded that encapsulation of catechins within niosomes is a promising approach to improve bioavailability by increasing stability and intestinal absorption [103]. 

#### 5.2.3. Solid Lipid Nanoparticles 

Solid lipid nanoparticles (SLNs) are particles composed of a solid lipid core, and were proposed to overcome certain limitations of liposomes and other nanoparticles [104]. Ramesh and Mandal constructed spherical SLNs as an EGCg carrier. The drug encapsulation efficiency was determined to be 81 ± 1.4%, and a sustained release of the encapsulated drug was noted. Unlike free EGCg, there was no sign of acute or sub-chronic toxicity from EGCg-loaded SLNs in a rat model. The pharmacokinetic and toxicokinetic data suggested that SLNs as an oral delivery system can enhance the stability and delivery of EGCg [105]. To improve the encapsulation efficiency of the lipid nanoparticles, Frias et al. designed nanostructured lipid carriers (NLCs) and compared their efficiency of encapsulating EGCg with that of SLNs. SLNs consist of only solid-phase lipids, while NLCs contain both solid- and liquid-phase lipids, resulting in a decreased crystallinity and therefore improved drug loading compared to SLNs [106]. As expected, the authors observed improved EGCg encapsulation in the NLCs (90%) compared to the SLNs (80%). Both formulations enabled the slow release of EGCg in simulated gastric and intestinal conditions and were demonstrated to be biocompatible [92]. Another study demonstrated that encapsulation within NLCs increased the stability of EGCg in both buffer and cell culture medium. A chitosan coating further improved this protective effect [107]. 

#### 5.2.4. Carbohydrate-Based Carriers 

To overcome degradation of the catechins as they travel through the gastrointestinal tract, Chung et al. coated catechins with hydroxypropyl methyl cellulose phthalate (HPMCP) coatings. The authors observed that the digestive stability, intestinal transport, and overall bioavailability were increased relative to noncoated catechins [108]. Similarly, encapsulation within γ-cyclodextrin increased the stability of the various catechin molecules in an in vitro digestion model and increased transport across a monolayer of Caco-2 cells, as a model of intestinal transport [109]. 

Xue et al. created EGCg nanocomplexes with glycosylated casein to enhance stability and bioavailability of EGCg. EGCg-glycosylated casein remained transparent and colloidally stable after 15 days of storage at 4 °C. The nanocomplex displayed a more controlled and sustained release of drug content than free EGCg when incubated in intestinal fluid because glycosylated casein was less susceptible to gastric enzyme degradation, providing exceptional stability to EGCg throughout the digestive system [89]. 

Dube et al. evaluated oral absorption effectiveness of EGCg encapsulated in chitosan-tripolyphosphate nanoparticles (CS NPs) [110]. CS NPs represent a highly stable, biodegradable, and biocompatible polymeric network appropriate for drug delivery and dietary use [90]. CS-encapsulation protected both C and EGCg from degradation in a slightly alkaline buffer [110]. CS NPs provided increased and sustained concentrations of EGCg in the gastrointestinal tract. Both in vitro and in vivo data showed that CS NPs enhanced the stability of EGCg through the gastrointestinal tract and allowed the complete release of EGCg in the jejunum [110]. EGCg-loaded CS NPs enhanced the plasma exposure of EGCg significantly in comparison to EGCg solution, suggesting that CS NPs provide a promising platform for the therapeutic application of EGCg and other polyphenolic compounds [110]. 

## 6. Conclusions

Catechins, both galloylated and nongalloylated, display a number of interesting properties, suggesting promising use in our treatment of bacterial infections. Whether as individual components or as a mixture with multiple components, catechins have demonstrated great therapeutic potential with promising experimental outcomes. Although catechins suffer from low bioavailability, recent research demonstrated that controlled delivery strategies have the potential to improve the in vivo effectiveness of this class of molecule. 

## Figures and Tables

**Table 1:** Antibacterial Properties of Catechins.

Bacterial Strains	Catechins	Concentration	Effects	Ref.
*P. aeruginosa* 10 clinical isolates ATCC 27853	EGCg GTE	≥0.4 mg/mL 8-fold dilution	Growth of all *P. aeruginosa* strains was inhibited.	[6]
*E. coli* 10 clinical isolates ATCC 25922	EGCg, GTE	≥0.4 mg/mL 4-fold dilution	Growth of all *E. coli* strains was inhibited.	[6]
*S. mutans*	EGCg	≥0.125 mg/mL	Inhibition of bacterial growth and biofilm formation.	[7]

**Table 2:** Anti-Virulence Properties of Catechins.

Virulence Factor	Catechins	Conc.	Effects	Ref.
*S. aureus α*-toxin	TPP		Inhibition of 82% of hemolytic activity.	[27]
*V. parahaemolyticus* thermostable direct hemolysin	TPP		Inhibition of 100% of hemolytic activity.	[27]
*V. cholerae* cholera toxin	EGCg	≥22 μM ≥22 μM	Host cell binding decreased by 60%. Activity of A subunit reduced by 70%.	[28]
*P. aeruginosa* exotoxin A	EGCg ECg	≥22 μM ≥23 μM	80% inhibition of cytotoxicity. 65% inhibition of cytotoxicity.	[28]
*Ricinus communis* ricin	EGCg	≥22 μM	44% inhibition of cytotoxicity.	[28]
*C. diphtheriae* diptheria toxin	EGCg	≥22 μM	50% inhibition of cytotoxicity.	[28]
*E. coli* Shiga-like toxins 1 and 2	EGCg, GCg	0.05 mg/mL	Inhibition of release of toxin	[29]
*E. coli* Shiga-like toxin 1	EGCg, GCg	15 mg/mL	Inhibition of cytotoxicity.	[30]
*B. anthracis* anthrax toxin	EGCg	97 nM	50% inhibition of metalloproteolytic activity.	[31]
*S. pneumoniae* pneumolysin	EGCg	≥1.09 μM	Inhibition of hemolytic activity	[2]
*L. monocytogenes* listeriolysin O	EGCg	≥10 nM	Inhibition of hemolytic activity and cholesterol binding.	[32]
*A. actinomycetemcomitans* leukotoxin	Cg, EGCg, GCg, ECg	≥1 mg/mL	Inhibition of cytotoxicity. Alterations in secondary structure.	[33,34]
*A. actinomycetemcomitans* outer membrane vesicles	EGCg	≥1 mg/mL	Inhibition of vesicle binding to host cells	[35]

## References

[1] U.S. Department of Health and Human Services, Centers for Disease Control and Prevention Antibiotic Resistance Threats in the United States, 2019.

[2] Song M., Teng Z., Li M., Niu X., Wang J., Deng X. (2017). Epigallocatechin gallate inhibits *Streptococcus pneumoniae* Virulence by Simultaneously Targeting Pneumolysin and Sortase A. J. Cell. Mol. Med..

[3] Gadkari V.P., Balaraman M. (2015). Catechins: Sources, Extraction and Encapsulation: A Review. Food Bioprod. Process..

[4] Botten D., Fugallo G., Fraternali F., Molteni C. (2015). Structural Properties of Green Tea Catechins. J. Phys. Chem. B.

[5] McNaught J.G. (1906). On the Action of Cold or Lukewarm Tea on *Bacillus typhosus*. J. R. Army Med. Corps.

[6] Jeon J., Kim J.H., Lee C.K., Oh C.H., Song H.J. (2014). The Antimicrobial Activity of (-)-Epigallocatehin-3-Gallate and Green Tea Extracts Against *Pseudomonas aeruginosa* and *Escherichia coli* Isolated from Skin Wounds. Ann Derm..

[7] Bai L., Takagi S., Ando T., Yoneyama H., Ito K., Mizugai H., Isogai E. (2016). Antimicrobial Activity of Tea Catechin Against Canine Oral Bacteria and the Functional Mechanisms. J. Vet. Med Sci..

[8] Sun Y., Hung W.-C., Chen F.-Y., Lee C.-C., Huang H.W. (2009). Interaction of Tea Catechin (-)-Epigallocatechin Gallate with Lipid Bilayers. Biophys. J..

[9] Tamba Y., Ohba S., Kubota M., Yoshioka H., Yoshioka H., Yamazaki M. (2007). Single GUV Method Reveals Interaction of tea Catechin (−)-Epigallocatechin Gallate with Lipid Membranes. Biophys. J..

[10] Tsuchiya H. (2001). Stereospecificity in Membrane Effects of Catechins. Chem.-Biol. Interact..

[11] Sirk T.W., Brown E.F., Sum A.K., Friedman M. (2008). Molecular Dynamics Study on the Biophysical Interactions of Seven Green tea Catechins with Lipid Bilayers of Cell Membranes. J. Agric. Food Chem..

[12] Singh N.B., Shankar S., Srivastava R.K. (2011). Green Tea Catechin, Epigallocatechin-3-Gallate (EGCg): Mechanisms, Perspectives and Clinical Applications. Biochem. Pharmacol..

[13] Sirk T.W., Brown E.F., Friedman M., Sum A.K. (2009). Molecular Binding of Catechins to Biomembranes: Relationship to Biological Activity. J. Agric. Food Chem..

[14] Kajiya K., Kumazawa S., Nakayama T. (2002). Effects of External Factors on the Interaction of Tea Catechins with Lipid Bilayers. Biosci. Biotechnol. Biochem..

[15] Ikigai H., Nakae T., Hara Y.T. (1993). Bactericidal Catechins Damage the Lipid Bilayer. Biochim. Et. Biophys. Acta (BBA) Biomembr..

[16] Arakawa H., Maeda M., Okubo S.T. (2004). Role of hydrogen Peroxide in Bactericidal Action of Catechin. Biol. Pharm. Bull..

[17] Kehrer J.P. (2000). The Haber–Weiss Reaction and Mechanisms of Toxicity. Toxicology.

[18] Imlay A.J., Linn S. (1986). Bimodal Pattern of Killing of DNA-Repair-Defective or Anoxically Grown *Escherichia coli* by Hydrogen Peroxide. J. Bacteriol..

[19] Shishido S., Miyano R., Nakashima T., Matsuo H., Iwatsuki M., Nakamura K., Kanno T., Egusa H., Niwano Y. (2018). A Novel Pathway for the Photooxidation of Catechin in Relation to its Prooxidative Activity. Sci. Rep..

[20] McClelland E.E., Bernhardt P., Casadevall A. (2006). Estimating the Relative Contributions of Virulence Factors for Pathogenic Microbes. Infect. Immun..

[21] Casadevall A., Pirofski L.-A. (2001). Host-Pathogen Interactions: The Attributes of Virulence. J. Infect. Dis..

[22] Cegelski L., Marshall G.R., Eldridge G.R., Hultgren S.J. (2008). The Biology and Future Prospects of Antivirulence Therapies. Nat. Rev. Microbiol..

[23] Clatworthy E.A., Pierson E., Hung D.T. (2007). Targeting Virulence: A New Paradigm for Antimicrobial Therapy. Nat. Chem. Biol..

[24] Mühlen S., Dersch P., Stadler M., Dersch P. (2016). Anti-Virulence Strategies to Target Bacterial Infections. How to Overcome the Antibiotic Crisis: Facts, Challenges, Technologies and Future Perspectives.

[25] Rasko A.D., Sperandio V. (2010). Anti-Virulence Strategies to Combat Bacteria-Mediated Disease. Nat. Rev. Drug Discov..

[26] Maura D., Ballok A.E., Rahme L.G. (2016). Considerations and Caveats in Anti-Virulence Drug Development. Curr. Opin. Microbiol..

[27] Okubo S., Ikigai H., Toda M., Shimamura T. (1989). The Anti-Haemolysin Activity of Tea and Coffee. Lett. Appl. Microbiol..

[28] Cherubin P., Garcia M.C., Curtis D., Britt C.B.T.J., Craft J.W., Burress H., Reddy S., Guyette J., Zheng T., Zheng T. (2016). et al. Inhibition of Cholera Toxin and Other AB Toxins by Polyphenolic Compounds. PLoS ONE.

[29] Sugita-Konishi Y., Hara-Kudo Y., Amano F., Okubo T., Aoi N., Iwaki M., Kumagai S. (1999). Epigallocatechin Gallate and Gallocatechin Gallate in Green Tea Catechins Inhibit Extracellular Release of Vero Toxin from Enterohemorrhagic *Escherichia coli* O157:H7. Biochim. Et. Biophys. Acta (BBA) Gen. Subj..

[30] Miyamoto T., Toyofuku S., Tachiki N., Kimura E., Zhou T., Ozawa T., Nakayama M., Shigemune N., Shimatani K., Tokuda H. (2014). Specific Inhibition of Cytotoxicity of Shiga-like Toxin 1 of Enterohemorrhagic *Escherichia Coli* by Gallocatechin Gallate and Epigallocatechin Gallate. Food Control.

[31] Dell’Aica I., Donà M., Tonello F., Piris A., Mock M.C. (2004). Potent Inhibitors of Anthrax Lethal Factor from Green Tea. EMBO Rep..

[32] Kohda C., Yanagawa Y., Shimamura T. (2008). Epigallocatechin Gallate Inhibits Intracellular Survival of *Listeria monocytogenes* in Macrophages. Biochem. Biophys. Res. Commun..

[33] Chang E.H., Huang J., Lin Z., Brown A.C. (2019). Catechin-Mediated Restructuring of a Bacterial Toxin Inhibits Activity. Biochim. Et. Biophys. Acta (BBA) Gen. Subj..

[34] Kawashima Y. (2011). Effects of Catechin Gallate on Bactericidal Action and Leukotoxic Activity of *Aggregatibacter actinomycetemcomitans*. Int. J. Oral-Med. Sci..

[35] Saito M., Tsuzukibashi O., Takada K. (2012). Anticytotoxic Effect of Green Tea Catechin on *Aggregatibacter actinomycetemcomitans* Vesicles. Int. J. Oral-Med. Sci..

[36] Toda M., Okubo S., Ikigai H., Shimamura T. (1990). Antibacterial and Anti-Hemolysin Activities of Tea Catechins and Their Structural Relatives. Nihon Saikingaku Zasshi. Jpn. J. Bacteriol..

[37] Ikigai H., Toda M., Okubo S., Hara Y., Shimamura T. (1990). Relationship Between the Anti-Hemolysin Activity and the Structure of Catechins and Theaflavins. Nihon Saikingaku Zasshi. Jpn. J. Bacteriol..

[38] Reddy S., Taylor M., Zhao M., Cherubin P., Geden S., Ray S., Francis D., Teter K. (2013). Grape Extracts Inhibit Multiple Events in the Cell Biology of Cholera Intoxication. PLoS ONE.

[39] Beddoe T., Paton A.W., Le Nours J., Rossjohn J., Paton J.C. (2010). Structure, Biological Functions and Applications of the AB5 Toxins. Trends Biochem. Sci..

[40] Collier R.J., Young J.A.T. (2003). Anthrax Toxin. Annu. Rev. Cell Dev. Biol..

[41] Tilley S.J., Orlova E.V., Gilbert R.J.C., Andrew P.W., Saibil H.R. (2005). Structural Basis of Pore Formation by the Bacterial Toxin Pneumolysin. Cell.

[42] Paterson K.G., Mitchell T.J. (2006). The Role of *Streptococcus Pneumoniae* Sortase a in Colonisation and Pathogenesis. Microbes Infect..

[43] Hamon M.A., Ribet D., Stavru F., Cossart P. (2012). Listeriolysin O: The Swiss Army Knife of Listeria. Trends Microbiol..

[44] Chang E.H., Giaquinto P., Huang J., Balashova N.V., Brown A.C. (2020). Epigallocatechin Gallate Inhibits Leukotoxin Release by *Aggregatibacter actinomycetemcomitans* by Promoting Association with the Bacterial Membrane. Mol. Oral Microbiol..

[45] Chang H.E., Brown A.C. (2021). Epigallocatechin Gallate Alters Leukotoxin Secretion and *Aggregatibacter actinomycetemcomitans* Virulence. J. Pharm. Pharmacol..

[46] Kachlany S.C., Fine D.H., Figurski D.H. (2000). Secretion of RTX Leukotoxin by *Actinobacillus actinomycetemcomitans*. Infect. Immun..

[47] Nice J.B., Balashova N.V., Kachlany S.C., Koufos E., Krueger E., Lally E.T., Brown A.C. (2018). *Aggregatibacter actinomycetemcomitans* Leukotoxin is Delivered to Host Cells in an LFA-1-Independent Manner when Associated with Outer Membrane Vesicles. Toxins.

[48] Kato S., Kowashi Y., Demuth D.R. (2002). Outer Membrane-Like Vesicles Secreted by *Actinobacillus actinomycetemcomitans* are Enriched in Leukotoxin. Microb. Pathog..

[49] Kulp A., Kuehn M.J. (2010). Biological Functions and Biogenesis of Secreted Bacterial Outer Membrane Vesicles. Annu. Rev. Microbiol..

[50] Ellis N.T., Kuehn M.J. (2010). Virulence and Immunomodulatory Roles of Bacterial Outer Membrane Vesicles. Microbiol. Mol. Biol. Rev..

[51] Kuehn J.M., Kesty C.N. (2005). Bacterial Outer Membrane Vesicles and the Host-Pathogen Interaction. Genes Dev..

[52] Demuth D., James D., Kowashi Y., Kato S. (2003). Interaction of *Actinobacillus Actinomycetemcomitans* Outer Membrane Vesicles with HL60 Cells Does not Require Leukotoxin. Cell. Microbiol..

[53] Sauvage E., Kerff F., Terrak M., Ayala J.A., Charlier P. (2008). The Penicillin-Binding Proteins: Structure and Role in Peptidoglycan Biosynthesis. FEMS Microbiol. Rev..

[54] Enright M.C. (2003). The Evolution of a Resistant Pathogen–the Case of MRSA. Curr. Opin. Pharmacol..

[55] Yam S.T., Hamilton-Miller J.M., Shah S. (1998). The Effect of a Component of Tea (Camellia Sinensis) on Methicillin Resistance, PBP2’ Synthesis, and Beta-Lactamase Production in *Staphylococcus aureus*. J. Antimicrob. Chemother..

[56] Hamilton-Miller T.J.M., Shah S. (2000). Activity of the Tea Component Epicatechin Gallate and Analogues Against Methicillin-Resistant *Staphylococcus aureus*. J. Antimicrob. Chemother..

[57] Zhao W.-H., Hu Z.-Q., Okubo S., Hara Y., Shimamura T. (2001). *;* Shimamura, T. Mechanism of Synergy Between Epigallocatechin Gallate and β-Lactams Against Methicillin-Resistant *Staphylococcus aureus*. Antimicrob. Agents Chemother..

[58] Stapleton P.D., Shah S., Anderson J.C., Hara Y., Hamilton-Miller J.M.T., Taylor P.W. (2004). Modulation of β-lactam Resistance in *Staphylococcus aureus* by Catechins and Gallates. Int. J. Antimicrob. Agents.

[59] Stapleton P.D., Shah S., Hara Y., Taylor P.W. (2006). Potentiation of Catechin Gallate-Mediated Sensitization of *Staphylococcus aureus* to Oxacillin by Nongalloylated Catechins. Antimicrob. Agents Chemother..

[60] Stapleton P.D., Shah S., Ehlert K., Hara Y., Taylor P.W. (2007). The Beta-Lactam-Resistance Modifier (-)-Epicatechin Gallate Alters the Architecture of the Cell Wall of *Staphylococcus aureus*. Microbiol (Read).

[61] Hajishengallis G., Liang S., Payne M.A., Hashim A., Jotwani R., Eskan M.A., McIntosh M.L., Alsam A., Kirkwood K.L., Lambris J.D. (2011). Low-Abundance Biofilm Species Orchestrates Inflammatory Periodontal Disease Through the Commensal Microbiota and Complement. Cell Host Microbe..

[62] Fournier-Larente J., Morin M.-P., Grenier D. (2016). Green Tea Catechins Potentiate the Effect of Antibiotics and Modulate Adherence and Gene Expression in *Porphyromonas gingivalis*. Arch. Oral Biol..

[63] Poole K. (2011). *Pseudomonas aeruginosa*: Resistance to the Max. Front. Microbiol..

[64] Betts J.W., Hornsey M., Higgins P.G., Lucassen K., Wille J., Salguero F.J., Seifert H., La Ragione R.M. (2019). Restoring the Activity of the Antibiotic Aztreonam Using the Polyphenol Epigallocatechin Gallate (EGCg) Against Multidrug-Resistant Clinical Isolates of *Pseudomonas aeruginosa*. J. Med. Microbiol..

[65] Sudano R.A., Blanco A.R., Giuliano F., Rusciano D., Enea V. (2004). Epigallocatechin-Gallate Enhances the Activity of Tetracycline in Staphylococci by Inhibiting its Efflux from Bacterial Cells. Antimicrob. Agents Chemother..

[66] Lee S., Razqan G.S.A., Kwon D.H. (2017). Antibacterial Activity of Epigallocatechin-3-Gallate (EGCg) and its Synergism with β-Lactam Antibiotics Sensitizing Carbapenem-Associated Multidrug Resistant Clinical Isolates of *Acinetobacter baumannii*. Phytomedicine.

[67] Seto R., Nakamura H., Nanjo F., Hara Y. (1997). Preparation of Epimers of Tea Catechins by Heat Treatment. Biosci. Biotechnol. Biochem..

[68] Sang S., Lee M.J., Hou Z., Ho C.T., Yang C.S. (2005). Stability of Tea Polyphenol (-)-Epigallocatechin-3-Gallate and Formation of Dimers and Epimers Under Common Experimental Conditions. J. Agric. Food Chem..

[69] Li N., Taylor L.S., Ferruzzi M.G., Mauer L.J. (2013). Color and Chemical Stability of Tea Polyphenol (−)-Epigallocatechin-3-Gallate in Solution and Solid States. Food Res. Int..

[70] Weisburger J.H. (1997). Tea and Health: A Historical Perspective. Cancer Lett..

[71] Chen Y.-C., Yu S.-H., Tsai G.-J., Tang D.-W., Mi F.-L., Peng Y.-P. (2010). Novel Technology for the Preparation of Self-Assembled Catechin/Gelatin Nanoparticles and their Characterization. J. Agric. Food Chem..

[72] Huang A., Honda Y., Li P., Tanaka T., Baba S. (2019). Integration of Epigallocatechin Gallate in Gelatin Sponges Attenuates Matrix Metalloproteinase-Dependent Degradation and Increases Bone Formation. Int. J. Mol. Sci..

[73] Dubey K.K., Janve M., Ray A., Singhal R.S., Galanakis C.M. (2020). Chapter 4-Ready-to-Drink Tea. Trends in Non-Alcoholic Beverages.

[74] Baell J.B. (2016). Feeling Nature’s Pains: Natural Products, Natural Product Drugs, and Pan Assay Interference Compounds (PAINS). J. Nat. Prod..

[75] Zorova L.D., Popkov V.A., Plotnikov E.Y., Silachev D.N., Pevzner I.B., Jankauskas S.S., Babenko V.A., Zorov S.D., Balakireva A.V., Juhaszova M. (2018). Mitochondrial Membrane Potential. Anal. Biochem..

[76] Kucera O., Mezera V., Moravcova A., Endlicher R., Lotkova H., Drahota Z., Cervinkova Z. (2015). In Vitro Toxicity of Epigallocatechin Gallate in Rat Liver Mitochondria and Hepatocytes. Oxid. Med. Cell. Longev..

[77] Lambert J.D., Kennett M.J., Sang S., Reuhl K.R., Ju J., Yang C.S. (2010). Hepatotoxicity of High Oral Dose (-)-Epigallocatechin-3-Gallate in Mice. Food Chem. Toxicol..

[78] Patel S.S., Beer S., Kearney D.L., Phillips G., Carter B.A. (2013). Green Tea Extract: A Potential Cause of Acute Liver Failure. World J. Gastroenterol..

[79] Chow H.-H.S., Hakim I.A., Vining D.R., Crowell J.A., Ranger-Moore J., Chew W.M., Celaya C.A., Rodney S.R., Hara Y.D.S. (2005). Effects of Dosing Condition on the Oral Bioavailability of Green Tea Catechins After Single-Dose Administration of Polyphenon e in Healthy Individuals. Clin. Cancer Res..

[80] Henning S.M., Choo J.J., Heber D. (2008). Nongallated Compared with Gallated Flavan-3-Ols in Green and Black Tea are More Bioavailable. J. Nutr..

[81] Leenen R., Roodenburg A.J.C., Tijburg L.B.M., Wiseman S.A. (2000). A Single Dose of Tea with or Without Milk Increases Plasma Antioxidant Activity in Humans. Eur. J. Clin. Nutr..

[82] Green R.J., Murphy A.S., Schulz B., Watkins B.A., Ferruzzi M.G. (2007). Common Tea Formulations Modulate in Vitro Digestive Recovery of Green Tea Catechins. Mol. Nutr. Food Res..

[83] Record I.R., Lane J.M. (2001). Simulated Intestinal Digestion of Green and Black Teas. Food Chem..

[84] Zhang L., Zheng Y., Chow M.S.S., Zuo Z. (2004). Investigation of Intestinal Absorption and Disposition of Green Tea Catechins by Caco-2 Monolayer Model. Int. J. Pharm..

[85] Vaidyanathan B.J., Walle T. (2001). Transport and Metabolism of the Tea Flavonoid (–)-Epicatechin by the Human Intestinal Cell Line Caco-2. Pharm. Res..

[86] Vaidyanathan J.B., Walle T. (2003). Cellular Uptake and Efflux of the Tea Flavonoid (-) Epicatechin-3-Gallate in the Human Intestinal Cell Line Caco-2. J. Pharmacol. Exp. Ther..

[87] Cai Z.Y., Li X.M., Liang J.P., Xiang L.P., Wang K.R., Shi Y.L., Yang R., Shi M., Ye J.H., Lu J.L. (2018). Bioavailability of Tea Catechins and its Improvement. Molecules.

[88] Babu S.N., Perumal C.S., Anjaneyulu V., Rajan D.S. (2019). Green Tea Catechin Loaded Nanodelivery Systems for the Treatment of Pandemic Diseases. Asian J. Pharm. Clin. Res..

[89] Xue J., Tan C., Zhang X., Feng B., Xia S. (2014). Fabrication of Epigallocatechin-3-Gallate Nanocarrier Based on Glycosylated Casein: Stability and Interaction Mechanism. J. Agric. Food Chem..

[90] Mohammed M.A., Syeda J.T.M., Wasan K.M., Wasan E.K. (2017). An Overview of Chitosan Nanoparticles and its Application in Non-Parenteral Drug Delivery. Pharmaceutics.

[91] Elsabahy M., Wooley K.L. (2012). Design of Polymeric Nanoparticles for Biomedical Delivery Applications. Chem. Soc. Rev..

[92] Frias I., Neves A.R., Pinheiro M., Reis S. (2016). Design, Development, and Characterization of Lipid Nanocarriers-Based Epigallocatechin Gallate Delivery System for Preventive and Therapeutic Supplementation. Drug Des. Dev. Ther..

[93] Koning A.G., Storm G. (2003). Targeted Drug Delivery Systems for the Intracellular Delivery of Macromolecular Drugs. Drug Discov Today.

[94] Hua S., Wu S.Y. (2013). The Use of Lipid-Based Nanocarriers for Targeted Pain Therapies. Front Pharm..

[95] Sercombe L., Veerati T., Moheimani F., Wu S.Y., Sood A.K., Hua S. (2015). Advances and Challenges of Liposome Assisted Drug Delivery. Front Pharm..

[96] Fang J.Y., Lee W.R., Shen S.C., Huang Y.L. (2006). Effect of Liposome Encapsulation of Tea Catechins on Their Accumulation in Basal Cell Carcinomas. J. Derm. Sci..

[97] Zou L.-Q., Peng S.-F., Liu W., Gan L., Liu W.-L., Liang R.-H., Liu C.-M., Niu J., Cao Y.-L., Liu Z. (2014). Improved in Vitro Digestion Stability of (−)-Epigallocatechin Gallate Through Nanoliposome Encapsulation. Food Res. Int..

[98] Huang Y.-B., Tsai M.-J., Wu P.-C., Tsai Y.-H., Wu Y.-H., Fang J.-Y. (2011). Elastic Liposomes as Carriers for Oral Delivery and the Brain Distribution of (+)-Catechin. J. Drug Target..

[99] Zou L.-Q., Liu W., Liu W.-L., Liang R.-H., Li T., Liu C.-M., Cao Y.-L., Niu J., Liu Z. (2014). Characterization and Bioavailability of Tea Polyphenol Nanoliposome Prepared by Combining an Ethanol Injection Method with Dynamic High-Pressure Microfluidization. J. Agric. Food Chem..

[100] Gharib A., Faezizadeh Z., Godarzee M. (2013). Therapeutic Efficacy of Epigallocatechin Gallate-Loaded Nanoliposomes Against Burn Wound Infection by Methicillin-Resistant *Staphylococcus aureus*. Ski. Pharmacol. Physiol..

[101] Fangueiro J.F., Calpena A.C., Clares B., Andreani T., Egea M.A., Veiga F.J., Garcia M.L., Silva A.M., Souto E.B. (2018). Biopharmaceutical Evaluation of Epigallocatechin Gallate-Loaded Cationic Lipid Nanoparticles (EGCg-LNs): In Vivo, in Vitro and Ex Vivo Studies. Stuart, A.; Pardakhty, G. Asadikaram and B. Poolman. Niosomes, an Alternative for Liposomal Delivery. PLoS ONE.

[102] Bartelds R., Nematollahi M.H., Pols T., Stuart M.C.A.A., Pardakhty G., Asadikaram B.P. (2018). Niosomes, an Alternative for Liposomal Delivery. PLoS ONE.

[103] Song Q., Li D., Zhou Y., Yang J., Yang W., Zhou G., Wen J. (2014). Enhanced Uptake and Transport of (+)-Catechin and (-)-Epigallocatechin Gallate in Niosomal Formulation by Human Intestinal Caco-2 Cells. Int. J. Nanomed..

[104] Müller H.R., Mäder K., Gohla S. (2000). Solid Lipid Nanoparticles (SLN) for Controlled Drug Delivery–a Review of the State of the Art. Eur. J. Pharm. Biopharm..

[105] Ramesh N., Mandal A.K.A. (2019). Pharmacokinetic, Toxicokinetic, and Bioavailability Studies of Epigallocatechin-3-Gallate Loaded Solid Lipid Nanoparticle in Rat Model. Drug Dev. Ind. Pharm..

[106] Muller H.R., Keck C.M. (2004). Challenges and Solutions for the Delivery of Biotech Drugs–a Review of Drug Nanocrystal Technology and Lipid Nanoparticles. J. Biotechnol..

[107] Zhang J., Nie S., Wang S. (2013). Nanoencapsulation Enhances Epigallocatechin-3-Gallate Stability and its Antiatherogenic Bioactivities in Macrophages. J. Agric. Food Chem..

[108] Chung J.-H., Lee S.-J., Chung J.-O., Oh Y.-J., Hwang J.-A., Kim Y.-K., Ko S., Shim S.-M. (2014). Effect of Hydroxypropyl Methyl Cellulose Phthalate Coating on Digestive Stability and Intestinal Transport of Green Tea Catechins. Integr. Med. Res..

[109] Son Y.-R., Chung J.-H., Ko S., Shimi S.-M. (2016). Combinatorial Enhancing Effects of Formulation and Encapsulation on Stability and Intestinal Transport of Green Tea Catechins. J. Microencapsul..

[110] Dube A., Nicolazzo J.A., Larson I. (2011). Chitosan Nanoparticles Enhance the Plasma Exposure of (−)-Epigallocatechin Gallate in Mice Through an Enhancement in Intestinal Stability. Eur. J. Pharm. Sci..

